# Feasibility of metacognitive interpersonal therapy treatments in European public services

**DOI:** 10.1192/j.eurpsy.2025.2153

**Published:** 2025-08-26

**Authors:** M. Pinotti, F. Inchausti, G. Dimaggio, R. Popolo, P. Ottavi

**Affiliations:** 1 South Psychiatric Operating Unit, Mental Health Department, Mental Health Center Rovereto, Trento, Italy; 2 University of La Rioja, La Rioja, Spain; 3 MIT Center, Rome, Italy

## Abstract

**Introduction:**

Metacognitive Interpersonal Therapy (MIT) is a third wave orientation psychotherapy, based on the understanding that severe mental illness features a combination of poor capacity to make sense of mental states or metacognition, maladaptive interpersonal schemas and dysfunctional coping procedures.

**Objectives:**

MIT is gathering increasing empirical support in both individual and group formats, in adults and adolescence in populations with personality disorders and early psychosis (Dimaggio et al., 2017; Fioravanti et al., 2024; Gordon-King et al., 2018; Pasetto et al., 2022; Inchausti et al., 2022; Popolo et al., 2018).

**Methods:**

The group format has been empirically tested in different countries (Italy, Spain and Norway).

**Results:**

Here we present the results of a series of RCT delivered in public mental health units (Ichausti et al., 2017; 2018; 2024; and Pinotti et al., 2024; Popolo et al., 2021; 2022).

**Conclusions:**

Overall results show that MIT in group can be successfully delivered in the context of public mental health facilities with evidence for its efficacy, feasibility and effectiveness.

**Disclosure of Interest:**

None Declared

